# Camp Sewers1Stenographic report of remarks made at the regular meeting of the Buffalo Sanatory Club, December 14, 1898.

**Published:** 1899-03

**Authors:** C. E. P. Babcock

**Affiliations:** 65th N. Y. V., Buffalo


### CAMP SEWERS.[1]

By MAJOR C. E. P. BABCOCK,

65th N. Y. V., Buffalo.

YOUR program has very properly divided or placed the subject of sewers under one head, and the disposal of sewage under another, which leaves very little to be said on the construction of sewers. There need be no argument as to the advisability of a permanent camp being properly sewered. I mean sewers being systematically built to carry everything from the kitchen and other sinks. With modern conveniences, such as the use of tiles of different sizes, the matter becomes a very simple one. The expense of construction in any ground available for a camp site would be small. For a 6-inch sewer from 23 to 30 cents per lineal foot; 9-inch, from 32 to 40 cents; 12-inch, 43 to 50 cents, so that the cost need be hardly considered at all. Taking this diagram as typical (from Infantry Drill Regulations, see Lieut. Harris's paper in February number) for a regiment, the necessary sewers we have mentioned would cost about $1,000. In case the camps have improved floors, that is, hard material, such as asphaltum or granolithic, the sewers would probably have to be somewhat larger than where the surface drainage would not be carried into them. If the tents were provided with plank floors and the company and battalion streets graded, any fall of rain would take care of itself. But if we have improved roads through the camp and hard material for floors, I think the drainage could be taken up by the sewers on account of the collection of dirt on the floors of the tents. That would involve the necessity of making a main sewer a trifle larger at very slight expense. I think that for a camp of this size, located almost anywhere in this state, such a system could be constructed for about $1,000. The estimated cost, as shown on the large plan, is $974; as shown on small plan, $710. Should water be supplied at head of company street, add about $110 to each estimate.

THE PRESIDENT—Can you estimate the time required to construct such a sewer?

MAJOR BABCOCK—From the time the material and men arrived on the ground, I should think possibly four days. If the men of the regiment had among their number a few who could lay the tile as they were delivered, the rest of the work could be carried on very quickly, and the regiment could construct it in about two days, provided the quartermaster would furnish picks and shovels.

1. Stenographic report of remarks made at the regular meeting of the Buffalo Sanatory Club, December 14, 1898.

